# Evidence of a causal relationship between blood pressure and pathological scars: a bidirectional Mendelian randomization study

**DOI:** 10.3389/fmed.2024.1405079

**Published:** 2024-07-24

**Authors:** Dan Du, Jiaqi Li, Xian Jiang

**Affiliations:** ^1^Department of Dermatology, West China Hospital, Sichuan University, Chengdu, China; ^2^Laboratory of Dermatology, Clinical Institute of Inflammation and Immunology, Frontiers Science Center for Disease-related Molecular Network, West China Hospital, Sichuan University, Chengdu, China; ^3^Med-X Center for Informatics, Sichuan University, Chengdu, China

**Keywords:** pathological scars, blood pressure, Mendelian randomization, keloid, hypertrophic scar, hypertension

## Abstract

**Background:**

Recent advancements in basic medicine and epidemiology suggest a potential influence of blood pressure on scar formation, yet the specifics of this relationship are not fully understood. This study aims to clarify the causal link between blood pressure and the development of pathological scars using Mendelian randomization (MR).

**Methods:**

This study employed genetic variants closely linked to blood pressure as instrumental variables to explore the relationship between blood pressure and pathological scars. The inverse variance weighted (IVW) method was used for analysis.

**Results:**

Our analysis identified a notable association where higher blood pressure was correlated with a lower risk of pathological scars. Specifically, an increase in diastolic blood pressure (odds ratio [OR] per standard deviation increase: 0.67 [95% Confidence Interval [CI], 0.49–0.99]), systolic blood pressure (OR per standard deviation increase: 0.66 [95% CI, 0.46–0.93]), and hypertension (pooled OR: 0.39 [95% CI, 0.18–0.85]) were significantly associated with a reduced risk of keloids. Similarly, a genetic predisposition to hypertension (pooled OR: 0.31 [95% CI, 0.11–0.89]) was significantly associated with a reduced risk of hypertrophic scars. Neither reverse MR analysis nor Steiger’s test indicated a significant reverse causal relationship between hypertension and either keloids or hypertrophic scars.

**Conclusion:**

The findings suggest a protective role of higher blood pressure against the development of pathological scars, including keloids and hypertrophic scars. However, the inconsistency observed across different MR methods warrants cautious interpretation and underscores the need for further investigation to confirm these findings.

## Introduction

Pathological scarring, encompassing keloids and hypertrophic scars, results from a variety of skin injuries such as trauma, burns, surgical incisions, irritation, and even insect bites (1). These scars develop due to damage to the dermis, triggering an abnormal wound healing process characterized by prolonged, histologically localized inflammation that affects the reticular dermis, leading to excessive inflammation and collagen deposition (2). Keloids are notorious for expanding beyond the original wound boundaries, whereas hypertrophic scars, though elevated and red, remain within the limits of the initial injury (3). The implications of these scars extend beyond aesthetic concerns, often causing functional impairments and psychological distress.

The ubiquity of trauma in daily life means that a wide swath of the population is susceptible to complications associated with pathological scarring. Globally, more than 100 million individuals experience issues related to pathological scars, and this statistic underscores not only the prevalence of scar-related health problems but also the complexity of managing a considerable number of these cases (4). The treatment difficulties faced by this subset highlight the need for advanced therapeutic strategies and a deeper understanding of scar pathology. Pathological scarring is influenced by a complex interplay of factors, including genetic predispositions, environmental influences, and possibly systemic health conditions, among which the influence of blood pressure on scar formation has recently come under scrutiny (1, 5). This influence may stem from an imbalance in the synthesis and breakdown of the extracellular matrix (ECM), hypoxia triggered by inflammation, and increased levels of angiotensin II (Ang II) (5–7). However, the genetic causal relationships between blood pressure and scar formation remain elusive, mainly due to confounding factors and the bidirectional nature of associations noted in observational studies.

Against this backdrop, Mendelian randomization (MR) has emerged as a novel tool that leverages genetic variants as instrumental variables to elucidate causal links, offering a new lens through which to examine the complex interplay between blood pressure and pathological scarring (8, 9). By using genetic variants for blood pressure, MR can shed light on the causative effects of blood pressure on pathological scars, free from the confounding factors endemic to traditional observational studies (10). This method is anchored in genetic epidemiological techniques, enhancing the robustness of analysis in the face of potential pleiotropy and other methodological challenges (9). Our investigation adopted a two-sample MR approach to elucidate this prospective causal link.

## Materials and methods

### Study design

**FIGURE 1:**
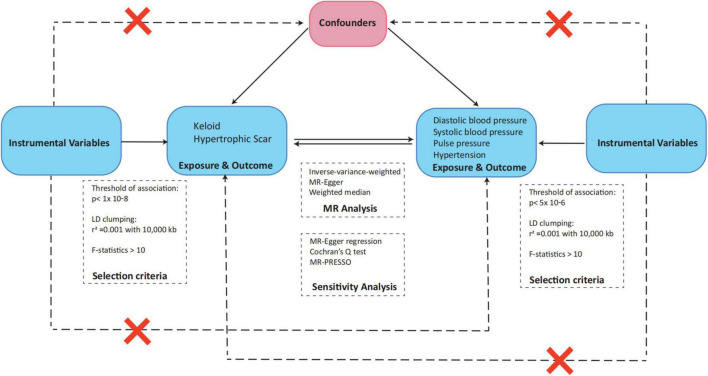
Overview of the Mendelian randomization analysis and three main assumptions.

We used a two-sample MR design to determine the possible causal effects between blood pressure-related events (diastolic blood pressure, systolic blood pressure, pulse pressure, hypertension) and pathologic scars (keloids and hypertrophic scars). Following the principles of MR, we formulated three fundamental hypotheses for our trial, each playing a crucial role in evaluating causality and revealing the connection between blood pressure and pathological scars. The first hypothesis, known as the relevance hypothesis, ensures that the genetic instruments we employed in our MR analysis accurately represent the intended exposure, enabling us to assess the impact of blood pressure-related events on the development of pathologic scars. The second hypothesis, referred to as the independence hypothesis, aims to confirm the independence of our genetic instruments from potential confounding factors, thereby minimizing the risk of false associations and strengthening the validity of our causal inferences. Finally, the third hypothesis, emphasizes the necessity of validating the assumption of exclusion restriction to establish a direct and unambiguous link between changes in blood pressure and the occurrence of pathologic scars. This validation further reinforces the causal interpretation of our MR analysis (Figure 1) (10).

### Data source

The information utilized for this MR investigation was obtained from various origins/studies Summary statistics obtained from published and publicly available GWAS were used in this study (Table 1). The large-scale GWAS data for diastolic blood pressure, systolic blood pressure, and pulse pressure used in this study were obtained from the meta-analysis conducted by Surendran et al. (11), which included 810,865 individuals of European ancestry. The GWAS data pertaining to hypertension were obtained from the UK Biobank and summarized by Dönertaş et al. (12). The GWAS data for keloids utilized in this analysis were obtained from a previous meta-analysis conducted by Sakaue et al. (13), and we specifically extracted data from the European population in this study, which included 668 keloid patients and 481,244 controls. The outcome data for hypertrophic scars were downloaded from the FinnGen website, which included 766 keloid patients and 207,482 controls.

**TABLE 1:** Baseline characteristics of included genome-wide association studies.

Trait	Consortium	Sample size	Ethnicity	Year	PubMed ID	IEU ID
Diastolic blood pressure	Meta	810,865	European	2020	33230300	ebi-a-GCST90000059
Systolic blood pressure	UK Biobank	810,865	European	2020	33230300	ebi-a-GCST90000062
Pulse pressure	UK Biobank	810,865	European	2020	33230300	ebi-a-GCST90000061
Hypertension	UK Biobank	484,598	European	2021	33959723	ebi-a-GCST90038604
Keloid	Meta	481,912	European	2021	34594039	ebi-a-GCST90018874
Hypertrophic scar	FinnGen	208,248	European	2021	/	finn-b-L12_HYPETROPHICSCAR

### Instrumental variable selection

To ascertain potential instrumental variables (IVs), we initiated the selection process by considering single-nucleotide polymorphisms (SNPs) associated with blood pressure related events at a genome-wide significance threshold of *p* < 1.0 × 10^–8^ and SNPs related to exposure from GWAS summary data of pathological scars at a genome-wide significance threshold of *p* < 5.0 × 10^–6^. The chosen IVs were required to satisfy specific quality control criteria: Initially, we established a linkage disequilibrium (LD) threshold for clustering at *r*^2^ < 0.001 and a window size of 10,000 kb to diminish the impact of LD on the outcomes. The selection of SNPs related to exposure from the GWAS summary data of the pathological scars at the genome-wide significance threshold of *p* < 5.0 × 10^–6^.

This measure aimed to ensure that the selected SNPs were relatively autonomous and not strongly correlated with one another. Subsequently, we standardized the impact estimations for both exposure and outcome variants, excluding any potential SNPs with incompatible alleles or palindromic characteristics. To maintain coherence, only SNPs accessible for all assessed traits were employed as IVs, with no surrogates employed to replace those missing in the outcome data. Next, each of the chosen SNPs was meticulously scrutinized using PhenoScanner V2.^1^ This tool offers comprehensive information on SNP phenotypes, aiding in the determination of whether the SNPs solely affect the outcomes through their exposure. Finally, to gauge the robustness of our selected instruments, we computed the F statistic using the formula: *F* = (β/SE)^2^, where β represents the magnitude of the effect and SE denotes the standard error of the effect magnitude, and a criterion of *F* > 10 was maintained, aligning with the principle of not displaying bias toward weak IVs (14).

### MR analysis

For our foundational analysis, we employed a suite of MR techniques to discern causal effects. This ensemble included the inverse-variance weighted (IVW) method, which served as our cornerstone, supplemented by the weighted median and MR-Egger methods. Together, these techniques facilitated a rigorous appraisal of causal connections. Heterogeneity between SNPs in the IVW and MR Egger analysis was evaluated using Cochran’s Q statistics. The intercept of MR-Egger regression was adapted to examine for predisposition from pleiotropy. This is a plausible supposition in this framework as no pleiotropic influence of the variant was observed after a search of all the SNPs, and the initiation of MR-Egger regression was not momentous. The median methodologies calculate the influence utilizing the median of the empirical distribution function of individual SNP ratio estimates and may furnish resilient calculations even if up to 50% of genetic variants are invalid instruments. An intercept term nearing zero indicates the absence of horizontal pleiotropy in the particular SNP under investigation in our bidirectional MR approach (15). To delve deeper into this phenomenon, we harnessed the MR- Pleiotropy RESidual Sum and Outlier (MR-PRESSO) global test, aiming to discern any horizontal pleiotropy where a singular genetic variant might influence an array of traits, muddling the causative assessments (16). Such tools shed light on the consistency and dependability of our findings. Finally, if exposures and outcomes were not amenable to reverse MR, MR Steiger’s test was initiated to robustly probe the direction between the exposure and the outcome. All analyses were performed using the package “Two-Sample-MR” (version 0.5.6), “MR-PRESSO” (version 1.0) in R (version 4.3.0).

## Results

In our research, we began by examining blood pressure-related events as exposure variables to explore their association with keloid and hypertrophic scars. IVs for our MR analysis were screened using a stringent *p*-value threshold of less than 1 × 10^–8^. This threshold was chosen to ensure the reliability of our IVs by prioritizing those with strong associations to the exposure.

**FIGURE 2:**
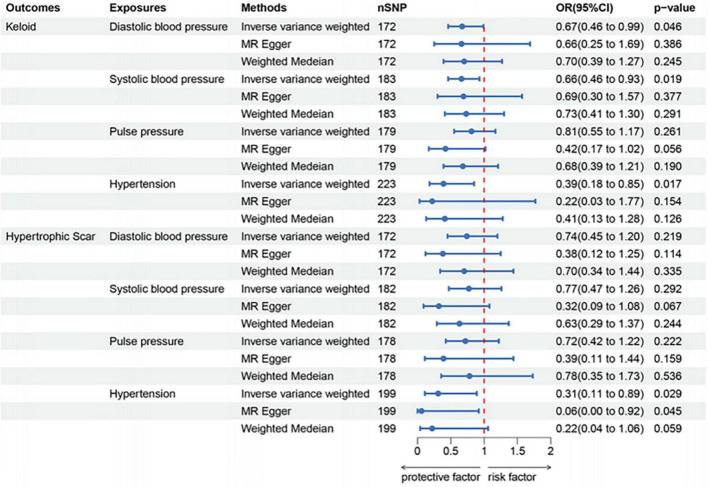
Forest plots of the association between blood pressure and pathological scars.

**FIGURE 3:**
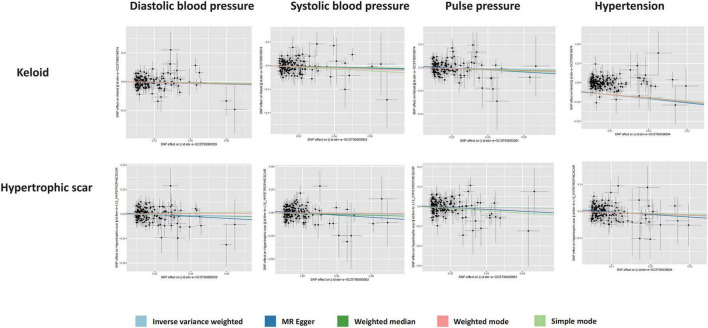
Scatter plots of the association between blood pressure and pathological scars.

Our findings revealed a notably reduced odds ratio (OR) in the IVW method for keloid, specifically for diastolic blood pressure (OR per standard deviation increase: 0.67 [95% Confidence Interval (CI), 0.46–0.99]), systolic blood pressure (OR per standard deviation increase: 0.66 [95% CI, 0.46–0.93]), and hypertension (pooled OR: 0.39 [95% CI, 0.18–0.85]). Additionally, a genetic predisposition to hypertension (pooled OR: 0.31 [95% CI, 0.11–0.89]) was significantly linked to a diminished risk of hypertrophic scars, as depicted in Figure 2. The association between genetically predicted pathological scarring and blood pressure-related events is detailed in Figure 3 through scatter plots.

To assess the variability in the associations between the exposures and outcomes, we applied two different MR methods: MR Egger and IVW. Our heterogeneity analysis, particularly the Q test, identified variability only in the relationship between diastolic blood pressure and keloids (Table 2). Although heterogeneity was identified in certain findings, the identified heterogeneity did not compromise the validity of the MR estimates as random-effect IVW in the present investigation, which could potentially equalize the pooled heterogeneity. In order to further examine the potential impact of alternative instrumental variables, we conducted supplementary analyses, encompassing MR Egger-intercept and MR-PRESSO, which collectively indicated the absence of horizontal pleiotropy in our study outcomes, proposing that no pleiotropic bias was introduced to MR estimates in the framework of heterogeneity (Table 3). Funnel plots also revealed no evidence of potential directional pleiotropy (Supplementary Figure 1).

**TABLE 2:** Heterogeneity results from the Cochran’s Q test of causal links between blood pressure and pathological scars.

Exposure	Outcome	IVW		MR-Egger	
		Q	*p*-value	Q	*p*-value
Diastolic blood pressure	Keloid	187	0.055	187	0.050
Systolic blood pressure		167	0.481	166	0.460
Pulse pressure		181	0.430	181	0.463
Hypertension		266	0.024*	226	0.025*
Diastolic blood pressure	Hypertrophic scar	138	0.870	157	0.876
Systolic blood pressure		161	0.585	158	0.627
Pulse pressure		170	0.644	170	0.643
Hypertension		233	0.222	230	0.246

*Significant heterogeneity.

**TABLE 3:** Pleiotropy results from Egger intercept analysis and MR-PRESSO.

Exposure	Outcome	MR egger-intercept		MR Presso global
		Intercept	*p*-value	
Diastolic blood pressure	Keloid	−0.001	0.902	0.054
Systolic blood pressure		−3.8e-4	0.960	0.473
Pulse pressure		0.012	0.112	0.481
Hypertension		0.005	0.548	0.045
Diastolic blood pressure	Hypertrophic scar	0.014	0.228	0.85
Systolic blood pressure		0.019	0.087	0.585
Pulse pressure		0.011	0.330	0.721
Hypertension		0.018	0.113	0.215

To determine the causality direction further, we performed a reverse MR analysis, with pathological scars as the exposure and blood pressure-related events as the outcome. It is important to note that MR analysis is not recommended with fewer than three IVs, and following these guidelines, we selected IVs filtered by a threshold of *p* < 5 × 10^–6^ for analysis. Our comprehensive results, employing various methodologies, consistently indicated no discernible reverse causal relationship between hypertension and either keloid or hypertrophic scar (Supplementary Table 1 and Supplementary Figure 2). For cases where reverse MR analysis was not feasible due to the limited number of IVs, we utilized MR Steiger directionality analysis to infer causality direction. The significance of all *p*-values being less than 0.05 suggests that our findings do not support the possibility of reverse causality (Supplementary Table 2).

## Discussion

This study introduces a counterintuitive hypothesis: elevated blood pressure might serve as a protective factor against keloids and hypertrophic scars, thus challenging prevailing assumptions.

By using MR, which employs genetic variants as tools to explore causal relationships, we emulate the randomization seen in controlled trials. This method helps us minimize the biases typically found in observational studies, bringing us a step closer to determining causality with greater clarity. In the stringent application of MR within our study, instrumental variables pertinent to hypertension were carefully selected from the expansive dataset of the UK Biobank, which comprises hundreds of thousands of clinical records. We applied more rigorous criteria (*p* < 1 × 10^–8^) than those typically utilized in MR studies, ensuring a robust association between these variables and blood pressure, thereby enhancing the credibility of our findings. The strength of MR, especially its capacity to isolate and account for confounders, helps explain why its conclusions may at times diverge from those drawn from conventional epidemiological data. Hypertension is correlated with various factors, including lifestyle choices, genetics, socioeconomic status, and environmental conditions, which can influence the development of pathological scars (17, 18). For instance, obesity has been implicated in the formation of scar keloids, and high salt intake is also associated with hypertrophic scar formation through mechanisms involving TRPC3-mediated mitochondrial Ca^2+^ homeostasis dysfunction (19, 20). By navigating through these confounding variables, our study offers a novel perspective that complements existing research and broadens our understanding of the relationship between blood pressure and pathological scars.

From a pathophysiological standpoint, current research posits that hypertension promotes the formation of pathological scars through a series of mechanisms, mainly including an imbalance in the synthesis and degradation of the ECM, inflammation-induced hypoxia, and elevated levels of Ang II (5–7). Initially, individuals with hypertension exhibit increased levels of tissue inhibitor of metalloproteinase-1 (TIMP-1), a key regulator of ECM structure (21, 22). This elevation mirrors that found in keloid fibroblasts, which suppress matrix metalloproteinase-1 (MMP-1), the enzyme responsible for breaking down collagen type I, thereby reducing ECM turnover (23, 24). These findings suggest that hypertension may intensify the ECM imbalance, creating an environment favorable for keloid development. Second, the role of hypertension in fibrosis is partially mediated by inflammation-induced tissue hypoxia. Inflammation increases the metabolic demands of cells, and concurrently, interstitial hypertension induces compression, resulting in decreased levels of metabolic substrates and ultimately leading to tissue hypoxia (5, 25). Hypoxia facilitates fibrogenesis *in vivo* through the induction of hypoxia-inducible factor-1 (HIF-1), a marker of local skin hypoxia, which then triggers epithelial-to-mesenchymal transition (EMT) (26). Moreover, HIF-1α triggers both the TGF-β/Smad and TLR4/MyD88/NF-κB signaling pathways, and the synergy between these pathways could facilitate keloid formation, and selective HIF-1α inhibitors consistently lead to a reduction in collagen levels (27, 28). Third, the renin-angiotensin system (RAS) plays a pivotal role in the regulation of blood pressure, extracellular fluid balance, and electrolyte homeostasis (29). Furthermore, activation of the RAS has been associated with fibrosis in various organs (30). Consequently, it is hypothesized that hypertension could influence the abnormal wound healing process, leading to skin fibrosis, primarily through the RAS.

However, the foundational evidence for some of these theories is not entirely conclusive. For instance, the direct connection between RAS abnormalities in pathological scars and hypertension is complex. A critical aspect of the RAS in pathological scars is the imbalanced expression ratio of local AT1 and AT2 receptors in skin tissue (31). The pro-fibrotic, pro-inflammatory, and pro-proliferative effects of Ang II are predominantly mediated through AT1 receptors, while AT2 receptors provide anti-fibrotic, anti-inflammatory, and anti-proliferative effects (32–34). AT1 receptor activation promotes keratinocyte and fibroblast migration and proliferation, increasing collagen production. These profibrotic effects are propagated through various pathways, including the IL-6/TGF-β and AP-1/TGF-β pathways, followed by the activation of SMAD 2/3, TAK1, and CTGF (32–36). Conversely, AT2 receptor stimulation hinders the activity of keratinocytes, fibroblasts, and collagen production, along with their respective signaling cascades (32, 37, 38). Keloid tissue shows elevated Ang II and AT1 receptor levels compared to normal skin and hypertrophic scars (39). However, the expression levels of angiotensin-converting enzyme (ACE) in the plasma of patients with keloids are not significantly different from those in individuals without keloids, and current evidence does not suggest any variations in the plasma levels of other components of the RAS between individuals with and without pathological scars (40). Moreover, although the hypothesis posits that hypertension-mediated, inflammation-induced tissue hypoxia is closely associated with the development of pathological scars, conclusive evidence regarding the differences in tissue oxygen concentrations between hypertensive and normotensive wound healing remains to be established. Hypertension can lead to endothelial dysfunction and abnormal inflammatory responses in wounds,(41, 42) but slight increases in blood pressure may theoretically enhance blood flow in certain areas, potentially boosting the oxygen supply. This phenomenon has been observed in animal studies, where hypertensive rats displayed a significant and immediate increase in blood flow compared to normotensive rats (43).

Further examination of the clinical literature revealed that the role of hypertension as a risk factor for pathological scarring may differ across populations. Recent UK Biobank-based research revealed a significant association between hypertension and the incidence of excessive scarring only among black individuals (44). Another study involving a Japanese cohort showed a correlation between high blood pressure and the size and number of keloids, although the prevalence of hypertension in keloid patients did not significantly differ from that in the general population (6). Interestingly, Japanese male keloid patients even had a lower hypertension rate than did the general male population (6). These findings indicate that, in populations other than those of African descent, the correlation between hypertension and the severity of pathological scars is more significant than that between hypertension and the incidence of pathological scars. Considering these outcomes, we hypothesize that ethnicity might act as a confounding factor with a modifier effect, potentially modifying the strength and direction of the relationship between hypertension and pathological scars. The effect of ethnicity as a modifier is not uncommon in medical research. For instance, research conducted by Jackson et al. (45) revealed that low-frequency, low-dose alcohol consumption acts as a protective factor against overall mortality in white men, yet it serves as a risk factor for black men. However, due to the predominance of European ancestry samples in the databases used, this hypothesis remains speculative within the confines of our MR analysis.

Our research has several limitations: First, although the IVW method was our primary tool for estimation, the results were not consistent across different MR methodologies. Second, despite excluding SNPs with significant pleiotropy during the selection of instrumental variables, we could not eliminate the impact of unrecognized confounders. Third, the GWAS data for keloids originates from a meta-analysis, where there may be some overlap between the sample information and that of hypertension-related events. Fourth, although we proposed a series of hypotheses regarding the relationship between hypertension and pathological scarring, these hypotheses have not been validated through basic experimental inflammation studies, which also provides direction for future research. Finally, the GWASs included in our study were all derived from individuals of European ancestry, limiting the generalizability of our findings to a wider population. This limitation also restricts our ability to test the hypothesis that ethnicity could serve as a confounding factor with a modifying effect, influencing both the magnitude and nature of the connection between blood pressure and pathological scars.

In summary, our research evaluated the causal relationship between blood pressure and pathological scarring using the MR approach, highlighting the intricate nature of the connection between blood pressure, wound healing, and pathological scarring. Rather than seeking to overturn or challenge the findings of previous studies, our MR study aims to provide a fresh perspective on this complex topic. From a clinical perspective, our research offers insights into whether active blood pressure management should be considered for patients with wounds to prevent pathological scarring.

## Data availability statement

The original contributions presented in this study are included in this article/Supplementary material, further inquiries can be directed to the corresponding author.

## Ethics statement

In accordance with local legislation and institutional requirements, ethical review and approval for the study involving human participants was not necessary. This article does not include any potentially identifiable images or data.

## Author contribuitons

DD: Writing – original draft, Writing – review and editing, Data curation, Methodology, Supervision, Conceptualization, Formal analysis, Project administration, Validation, Investigation, Funding acquisition, Resources, Visualization, Software. JL: Conceptualization, Data curation, Formal analysis, Funding acquisition, Investigation, Methodology, Project administration, Resources, Software, Supervision, Validation, Visualization, Writing – original draft, Writing – review & editing. XJ: Conceptualization, Data curation, Formal analysis, Funding acquisition, Investigation, Methodology, Project administration, Resources, Software, Supervision, Validation, Visualization, Writing – original draft, Writing – review & editing.

## Footnotes

1
http://www.phenoscanner.medschl.cam.ac.uk/

## Supplementary material

Supplementary Figure 1 Funnel plots of the causal association between blood pressure and pathological scars.

Supplementary Figure 2 Scatter plots and funnel plots of the causal association between pathological scars and hypertension.
