# Supplementary material for: Evidence of a causal relationship between blood pressure and pathological scars: a bidirectional Mendelian randomization study
Source: Front Med (Lausanne). 2024 Jul 24;11:1405079. doi: 10.3389/fmed.2024.1405079 (PMC11303301; doi:10.3389/fmed.2024.1405079)
Supplement: Supplementary file 1 [file Table_1.docx]

**Supplementary Table 1. Reverse MR results of causal links between pathological scars and hypertension.**

**MR, Mendelian randomization**

| **Exposure** | **Outcome** | **Methods** | **nSNP** | **OR(95%CI)** | **P value** |
| --- | --- | --- | --- | --- | --- |
| Keloid | Hypertension | Inverse variance weighted | 19 | 1.00 (1.00, 1.00) | 0.098 |
|  |  | MR Egger | 19 | 1.00 (1.00, 1.01) | 0.475 |
|  |  | Weighted median | 19 | 1.00 (1.00, 1.00) | 0.686 |
| Hypertrophic scar |  | Inverse variance weighted | 9 | 1.00 (1.00, 1.00) | 0.412 |
|  |  | MR Egger | 9 | 1.00 (1.00, 1.01) | 0.392 |
|  |  | Weighted median | 9 | 1.00 (1.00, 1.00) | 0.857 |

**Supplementary Table 2. Directionality test results form Steiger’s test of causal links between blood pressure and pathological scars**

| **Exposure** | **Outcome** | **Steiger’s test**  **p value** |
| --- | --- | --- |
| Diastolic blood pressure | Keloid | 0 |
| Systolic blood pressure |  | 0 |
| Pulse pressure |  | 0 |
| Hypertension |  | 0 |
| Diastolic blood pressure | Hypertrophic scar | 0 |
| Systolic blood pressure |  | 0 |
| Pulse pressure |  | 0 |
| Hypertension |  | 0 |
