# Supplementary figures and images for: Evidence of a causal relationship between blood pressure and pathological scars: a bidirectional Mendelian randomization study
Source: Front Med (Lausanne). 2024 Jul 24;11:1405079. doi: 10.3389/fmed.2024.1405079 (PMC11303301; doi:10.3389/fmed.2024.1405079)

Keloids

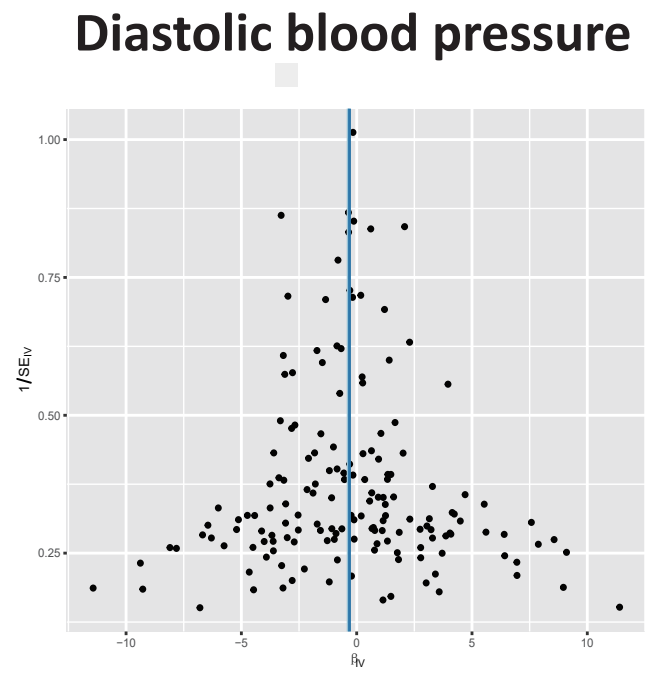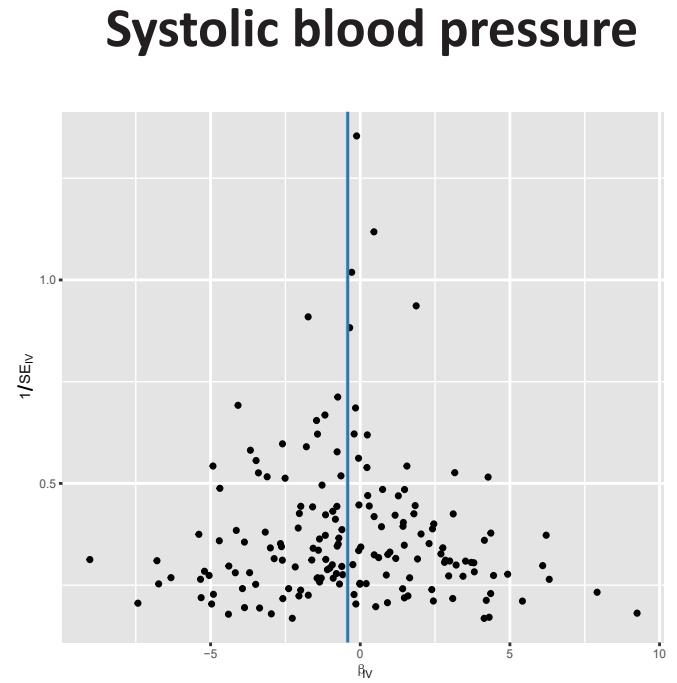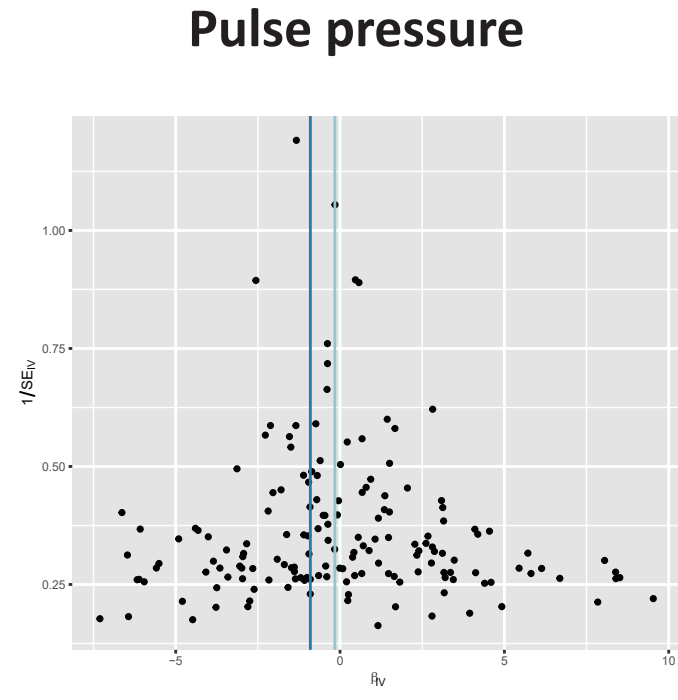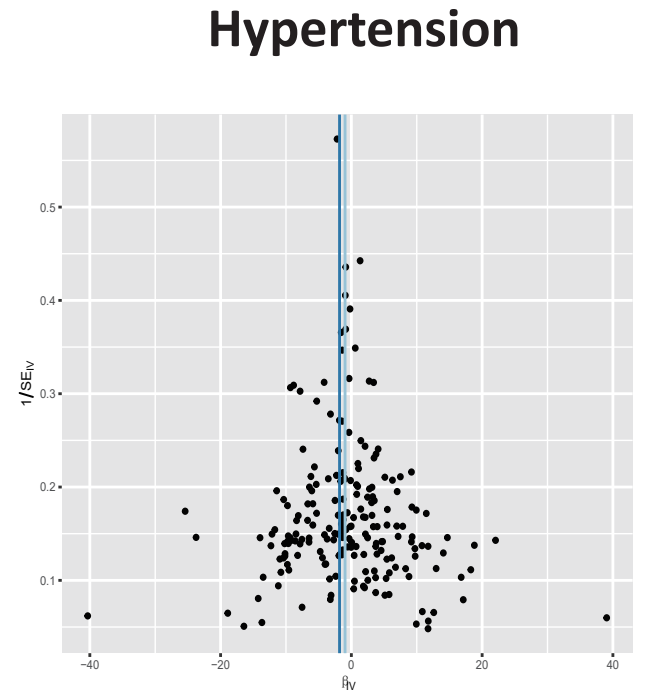

Hypertrophic scars

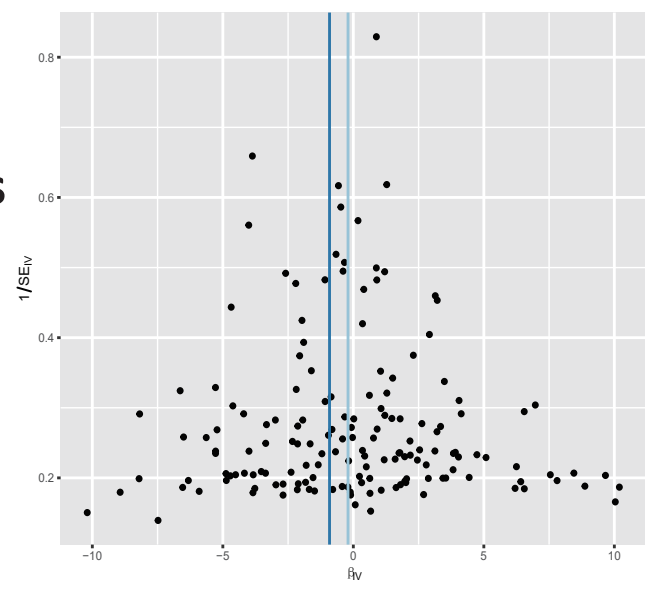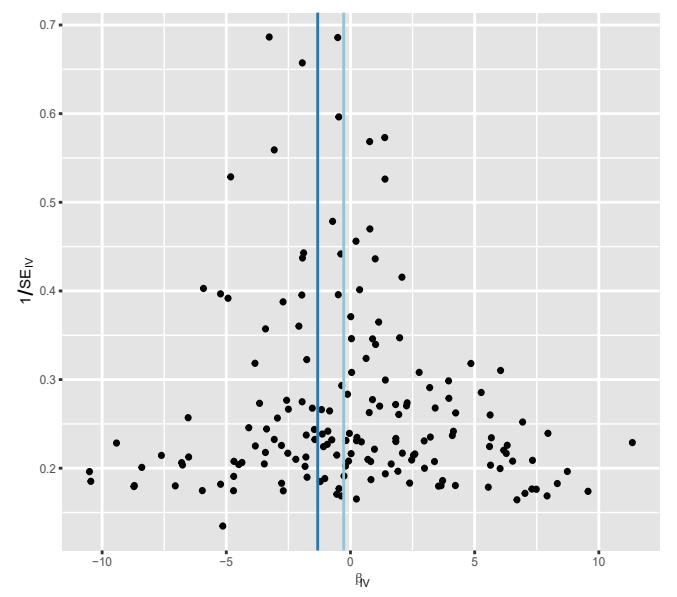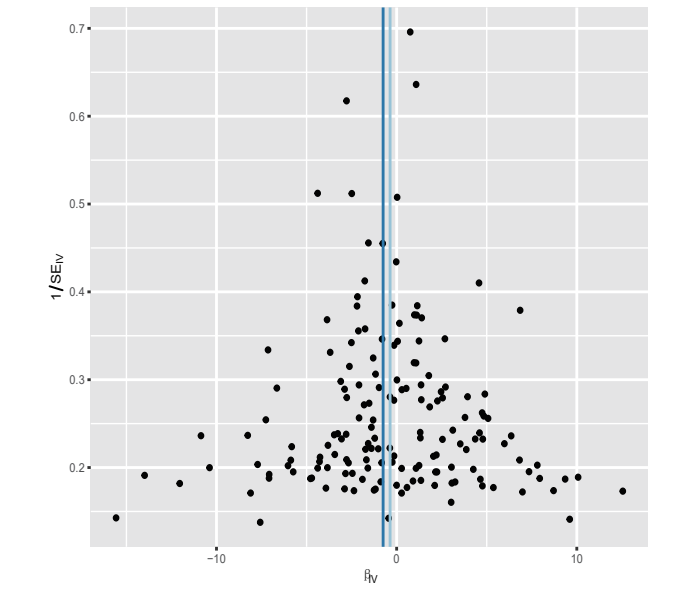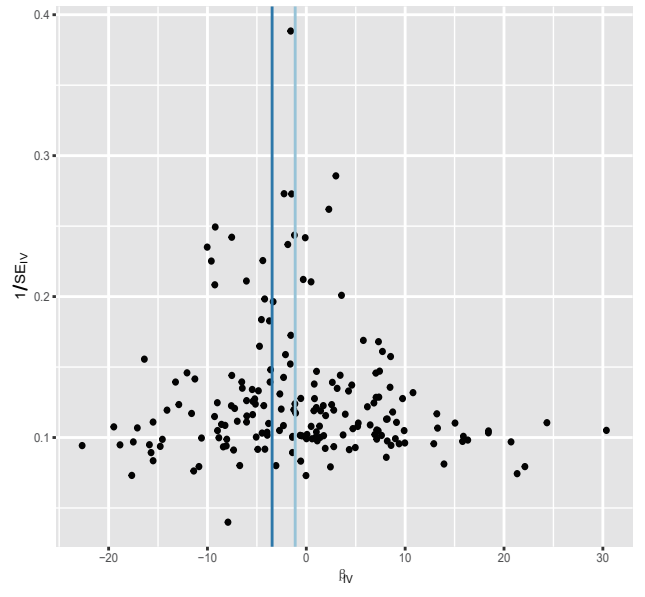

MR Egger

Inverse variance weighted

Supplement: Supplementary Figure 1 — Funnel plots of the causal association between blood pressure and pathological scars. [file Data_Sheet_1.PDF]
