# Supplementary material for: Evidence of a causal relationship between blood pressure and pathological scars: a bidirectional Mendelian randomization study
Source: Front Med (Lausanne). 2024 Jul 24;11:1405079. doi: 10.3389/fmed.2024.1405079 (PMC11303301; doi:10.3389/fmed.2024.1405079)
Supplement: Supplementary Figure 2 — Scatter plots and funnel plots of the causal association between pathological scars and hypertension. [file Data_Sheet_2.PDF]

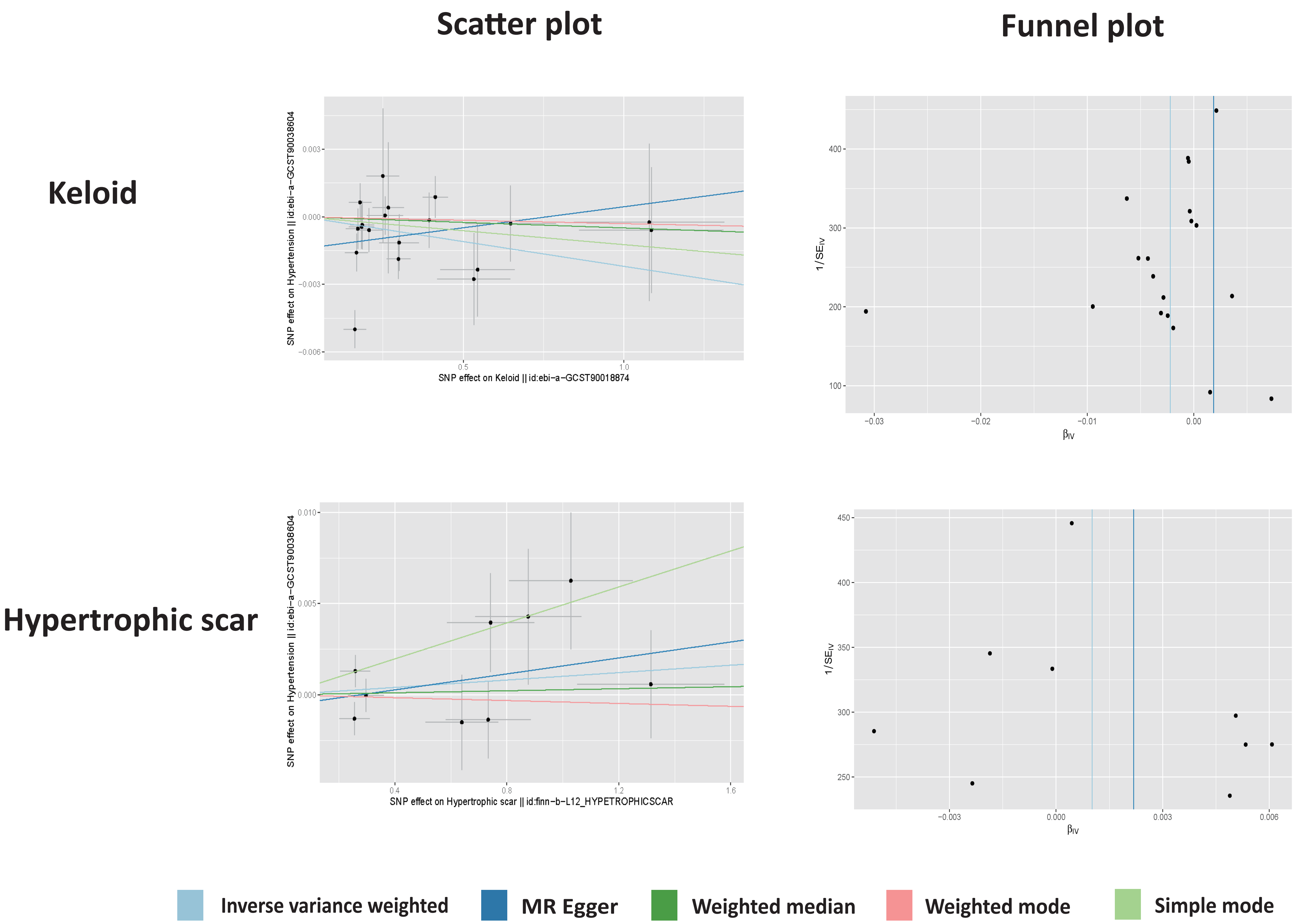
Scatter plot
Funnel plot
Keloid
Hypertrophic scar
SNP effect on Hypertension || id:ebi-a-GCST90038604
SNP effect on Keloid || id:ebi-a-GCST90018874
SNP effect on Hypertrophic scar || id:finn-b-L12_HYPETROPHICSCAR
1/SEIV
βIV
Inverse variance weighted
MR Egger
Weighted median
Weighted mode
Simple mode
